# Synthesis and biological evaluation of novel quinoline analogs of ketoprofen as multidrug resistance protein 2 (MRP2) inhibitors

**DOI:** 10.22038/ijbms.2021.54554.12265

**Published:** 2021-06

**Authors:** Fatemeh Mosaffa, Farzin Hadizadeh, Faezeh Fathi, Zahra Eslami Nasab, Tahereh Pourzahed, Sayyed Mohammad Aboutorabzade, Razieh Ghodsi

**Affiliations:** 1 Biotechnology Research Center, Institute of Pharmaceutical Technology, Mashhad University of Medical Sciences, Mashhad, Iran; 2 Department of Medicinal Chemistry, School of Pharmacy, Mashhad University of Medical Sciences, Mashhad, Iran

**Keywords:** Anticancer, (ATP)-binding cassette, Multidrug resistance – protein, Multidrug resistance - protein inhibitor, Molecular docking, Quinoline, Synthesis

## Abstract

**Objective(s):**

A new series of quinoline analogs of ketoprofen was designed and synthesized as multidrug resistance protein 2 (MRP2) inhibitors using ketoprofen as the lead compounds.

**Materials and Methods:**

The cytotoxic activity of the compounds was evaluated againt two cancer cell lines including A2780/RCIS (MRP2-overexpressing ovarian carcinoma), A2780, drug-sensitive ovarian carcinoma using MTT assay. Compounds showing low toxicity in MTT test were selected to investigate their MRP inhibition activity. MRP2 inhibitory potency was evaluated by determination of the uptake amount of fluorescent 5-carboxy fluorescein diacetate (5-CFDA) substrate, by A2780/RCIS in the presence of the selected compounds. Mode of interaction between synthesized ligands and homology modeled MRP2 was investigated by MOE software.

**Results:**

Compound **6d**, a 4-carboxy quinoline possessing dimethoxy phenyl in position 2 of quinoline ring, showed the most MRP2 inhibition activity among all the quinolines and more than the reference drug ketoprofen. MRP2 inhibition activity of compound **7d** was less in comparison to that of compound **6d**, indicating that carboxyl group in position 4 of quinoline may interact with MRP2. Docking studies showed that compound **7d** methyl ester of **6d**, interacted less compared to its parent **6d**, which is consistent with biological results.

**Conclusion:**

This study indicates that 6- or 8-benzoyl-2-arylquinoline is a suitable scaffold to design MRP2 inhibitors. The position of benzoyl in quinoline ring is important in inhibition of MRP2. Generally, MRP2 inhibition activity of compound **7d** was less in comparison to that of **6d**, indicating that carboxyl group in position 4 of quinoline may interact with MRP2.

## Introduction

Cancer is the reason of 25% of all deaths in developed countries (1). Although chemotherapy is the collective way for treatment of different cancers, it fails to treat most cancer patients with advanced disease due to the occurrence of drug resistance (2, 3). One of the most essential mechanisms underlying MDR (multidrug resistance) is the overexpression of adenosine triphosphate (ATP)-binding cassette (ABC) super-family of transporters, which efflux both cytotoxic agents and targeted anticancer drugs using ATP driven energy (4). One important class of the ABC family is the human multidrug resistance-associated protein (MRP) family which comprises seven members. Numerous members of the MRP family especially MRP1 and MRP2 are complicated in the detoxification and defense of the host against xenobiotic materials. They are also expected to cause drug resistance by their ability in moving a wide range of anticancer drugs out of the cells and their occurrence in many different types of cancers (5).

NSAIDs (Non-steroidal anti-inflammatory drugs) have been administrated as analgesic, antipyretic and anti-inflammatory agents for several years (6, 7). NSAIDs also have been widely considered for their anti-tumorigenic and chemosensitive properties (8, 9). In addition, it was described that aspirin and indomethacin had anti-proliferative and anti-MDR activities (10, 11). Enhancements of anticancer drugs cytotoxicity in multidrug resistant cancer cells by NSAIDs were also reported by researchers (12-21). So, NSAIDs were thought to have the potential to be antitumor and chemosensitive agents for cure of some MDR cancers (22).

Some authors have distinguished that NSAIDs can enhance antitumor activity of drugs, working as inhibitors of multidrug resistance proteins MRP or MDR1 (15, 23). Enforced expression of COX-2 causes enhancement in MDR1 expression, so the use of COX-2 inhibitors to decrease action of MDR1 may enhance accumulation of chemotherapy agents and reduce resistance of tumors to anticancer drugs. It has already been described that NSAIDs inhibit MRP2 or MRP4 (24, 25). Also, NSAIDs such as salicylate, piroxicam, ibuprofen, naproxen, sulindac, tolmetin, etodolac, dicrofenac, indomethacin, ketoprofen, phenylbutazone and celecoxib inhibit MRP1, MRP2 and/or MRP4 (25, 26). A wide variety of NSAIDs like indomethacin and ketoprofen inhibited MRP2 and MRP4 facilitated methotrexate transport at concentrations to which the transporters may be unprotected under therapeutic conditions. As ketoprofen is a well-known MRP2 inhibitor (14) and some quinoline derivatives such as quinine (27-31) reported as MRP modulators, we designed novel 2-(aryl)quinolines possessing ketoprofen scaffold as MRP2 inhibitors. The rational for the design of these compounds is represented in Figure 2. The cytotoxic activity of the synthesized compounds was evaluated against two human cancer cell lines including A2780/RCIS, cisplatin resistant human ovarian carcinoma (MRP2-overexpressing ovarian carcinoma); A2780, drug-sensitive ovarian carcinoma. Compounds showed low to moderate toxicity in MTT test were selected to investigate their MRP 2 inhibition activity. Moreover, trying to explain the results of biological experiments, docking studies of the selected compounds into the homology-modeled human MRP2, were carried out.

## Materials and Methods

***Chemistry***

All reagents, chemicals and solvents used in this research were bought from Merck AG and Aldrich Chemical. Melting points were assessed using a Thomas–Hoover capillary apparatus. Infrared spectra were attained by a Perkin Elmer (Model 1420) spectrometer. To acquire ^1^HNMR spectra Bruker FT-500 and 300 MHz instruments (Brucker Biosciences, USA) was used and A Bruker FT-300 MHz instrument was used to obtain ^13^CNMR spectra. Chloroform-D and DMSO-D6 were used as solvents. Coupling constant (J) values are measured in hertz (Hz) and spin multiples are given as s (singlet), d (double), t (triplet), q (quartet), m (multiplet). The mass spectra were assessed using a 3200 QTRAP LCMS triple quadrupole mass spectrometer (LCMS) with an electrospray ionization (ESI) interface. 

***General procedure for preparation of 6- or 8-benzoyl-2-arylquinoline-4-carboxylic acid (Doebner reaction)***

A solution of appropriate benzaldehyde (9.45 mmol) and pyruvic acid (1.25g, 14.3 mmol) in acetic acid (10 ml) was heated for 40 min then 2- or 4-aminobenzophenone (10 mmol) was added to the solution and refluxed overnight. After cooling, the formed precipitate was filtered and washed with hexane and recrystallized in ethanol.

***8-Benzoyl-2-phenylquinoline-4-carboxylic acid (4a)***

Yield: 25%; mp=247-249 °C; ^1^H NMR (300MHz-DMSO-d_6_): δ (ppm)7.34-7.45 (m, 3H, phenyl H_3_&H_4_&H_5_), 7.48-7.51(t, 2H, benzoyl H_3_&H_5_, *J*=9Hz),7.65-7.68 (t, 1H, benzoyl H_4_, *J*=9 Hz), 7.70-7.79 (m,4H, benzoyl H_2_&H_6_& phenyl H_2_&H_6_), 7.83-7.86 (t, 1H, quinoline H_6_, *J*=9 Hz),7.97-8.0 (dd,1H,quinoline H_5_, *J*=9Hz*, J*=2.5Hz), 8.5 (s,1H, quinoline H_3_), 8.84-8.87 (dd, 1H, quinoline H_7_, *J*=9Hz, *J*=2.5Hz), 13.05 (s, 1H, COOH); ^13^C NMR (DMSO-d_6_, 75 MHz): δ 119.65, 119.98, 123.74, 126.77, 127.31, 127.96, 128.51, 127.81 , 128.66, 130.08 , 131.26, 130.39, 137.74, 138.33, 138.95, 139.56, 155.83, 167.87, 198.01; LC-MS(ESI) :352.0 (M-1).

***8-benzoyl-2-(4-fluorophenyl)quinoline-4-carboxylic acid (4b)***

Yield: 73%; mp=183-185 °C; IR (KBr): ν (cm^-1^) 3002.55 (OH) 1691.63, 1659.10 (C=O); ^1^H NMR (300MHz-DMSO-d_6_): δ (ppm)7.21 -7.24 (t, 2H, 4-flurophenyl H_3_&H_5_, *J*=9 Hz), 7.48-7.53 (t, 2H, 4-flurophenyl H_2_&H_6, _*J*=9 Hz), 7.62-7. 65 (t, 1H, phenyl H_4_, *J*=9 Hz), 7.67-7.73(dd, 2H, phenyl H_2_&H_6_, *J*=9Hz, *J*=2.5 Hz), 7.79-7.88 (m, 3H, quinoline H_6_& phenyl H_3_&H_5_), 7.97-8.0 (dd, 1H, quinoline H_5_, *J*=9Hz, J=2.5 Hz), 8.49 (s, 1H, quinoline H_3_), 8.81-8.84 (dd,1H, quinoline H_7_, J=9Hz), 14.16 (s, 1H, COOH); ^13^C NMR (DMSO-d_6_, 75 MHz): δ116.06,116.35,119.65,123.63,127.98,128.07 ,129.07,129.64, ,129.81, 133.54 ,134.25,134.29,138.45,138.94,139.46,146.28,154.81, 167.80,197.92; LC-MS(ESI):370.0(M-1).

***8-Benzoyl-2-(p-tolyl)quinoline-4-carboxylic acid (4c)***

Yield: 33%; mp=252-254 °C; IR (KBr): ν (cm^-1^) 2865.68 (OH) 1701.50, 1659.41 (C=O); ^1^H NMR (300MHz-DMSO-d_6_):δ 2.30 (s, 3H, methyl), 7.15-7.17 (d, 2H, 4-methyl phenylH_3_&H_5_*, J*=6Hz), 7.47-7.50 (t, 2H, phenyl H_3_&H_5_, *J*=9Hz), 7.62-7.67(m, 5H, phenyl H_2_&H_6_& H_4 _&4-methyl phenyl H_2_&H_6_), 7.81-7.84 (t, 1H, quinolineH_6_, J=9Hz), 7.96-7.99 (dd, 1H, quinoline H_5_, J=9Hz, J=2.5Hz), 8.46(s, 1H, quinoline H_3_), 8.82-8.85(dd, 1H, quinoline H_7_, J=9Hz, J=2.5Hz), 14.13(s, 1H, COOH); ^13^C NMR (DMSO-d_6_, 75 MHz): δ 21.27, 119.61, 123.61, 127.38, 127.76, 128.09, 129.01,129.63, 129.72, 129.88, 133.44, 135.01, 138.16, 139.05, 139.45, 140.55, 146.40, 155.78, 167.87, 198.09 ;LC-MS(ESI): 366.2 (M-1).

***8-Benzoyl-2-(3,4-dimethoxyphenyl)quinoline-4-carboxylic acid (4d)***

Yield: 22%; mp=266-268 °C; IR (KBr): ν (cm^-1^) 2960.9 (OH) 1700.7, 1667.8 (C=O); ^1^H NMR (300MHz-DMSO-d_6_):δ (ppm) 3.48(s, 3H, OCH_3_), 3.31(s, 3H, OCH_3_), 6.95-6.98 (d, 1H, 3&4-dimethoxyphenyl H_3_, *J*=9Hz), 7.22 (s,1H, 3&4-dimethoxyphenyl H_6_), 7.46-7.49 (dd, 2H, phenyl H_3_&H_5_, *J*=9Hz, *J=*2.5Hz), 7.54-7.57 (dd, 1H, 3&4-dimethoxyphenyl H_2_, *J*=9Hz, *J=*2.5Hz), 7.60-7.63 (t, 1H, quinoline H_4_ , *J*=9Hz), 7.73-7.76 (dd, 2H, phenyl H_2_&H_6_, *J*=9Hz, *J*=2.5Hz), 7.79-7.82 (t, 1H, quinolineH_6_, *J*=9Hz), 7.94-7.97 (dd, 1H, quinoline H_5_, *J*=9Hz, *J=*2.5Hz), 8.47(s, 1H, quinolineH_3_), 8.78-8.81(dd, 1H, quinoline H_7_*, J*=9Hz, *J=*2.5Hz), 13.9 (s, 1H, COOH); ^ 13^C NMR (DMSO-d_6_, 75 MHz): δ 55.65, 56.01 ,109.87, 111.88, 119.42, 120.66, 123.29, 127.55, 129.06, 129.63, 129.73, 130.27, 133.55, 138.12, 138.71, 139.43, 149.37, 151.28, 155.46, 167.95, 198.10; LC-MS(ESI):412.0 (M-1)

***6-Benzoyl-2-phenylquinoline-4-carboxylic acid (6a)***

Yield: 26%; mp=226-228 °C; IR (KBr): ν (cm^-1^) 3055(OH) 1700.7, 1663.1 (C=O); ^1^H NMR (300MHz-DMSO-d_6_):δ (ppm)7.57-7.64 (m, 5H, phenyl), 7.74-7.77 (t, 1H, benzoyl H_3_, *J*=9Hz), 7.84-7.87 (dd, 2H, benzoyl H_2 _&H_6_, *J*=9Hz, *J*=2.5Hz), 8.14-8.17(dd, 1H, quinolineH7*, J*=9Hz, *J*=2.5Hz), 8.26-8.29(d, 1H, quinoline H_8_, *J*=9Hz), 8.32-8.35(dd, 2H, benzoyl H_3 _&H_5_, *J*=9Hz), 8.57 (s, 1H, quinoline H_3_), 8.14 (s, 1H, quinoline H_5_), 14.10 (s, 1H, COOH);^ 13^C NMR (DMSO-d_6_, 75 MHz): δ 120.81, 123.12, 127.94, 129.09, 129.52, 130.02, 130.28, 130.50, 130.68, 130.99, 133.36, 135.68, 137.33, 137.92, 138.81, 150.31, 158.45, 167.49, 195.70; LC-MS(ESI):352.0 (M-1).

***6-Benzoyl-2-(4-fluorophenyl)quinoline-4-carboxylic acid (6b)***

Yield: 16%; mp=268-270 °C; IR (KBr): ν (cm^-1^) 3059.6 (OH) 1705.4, 1658.4 (C=O); ^1^H NMR (300MHz-DMSO-d_6_):δ (ppm)7.41-7.44(t, 2H, phenyl H_3 _&H_5_, *J*=9Hz), 7.61-7.64(t, 2H, 4-flurophenyl H_3 _&H_5_, *J*=9Hz), 7.74-7.77 (t, 1H, phenylH_4_, *J*=9Hz), 7.85-7.88 (d, 2H, phenyl H_2 _&H_6_*, J*=9Hz), 8.13-8.16 (dd, 1H, quinoline H_7_,* J*=9Hz, *J*=2.5Hz), 8.23-8.26 (d, 1H, quinoline H_8_, *J*=9Hz), 8.40-8.43 (dd, 2H, 4-flurophenyl H_2 _&H_6_, *J*=9Hz, *J=*2.5Hz), 8.54 (s, 1H, quinoline H_3_), 9.11(s, 1H, quinoline H_5_), 14.04(s, 1H, COOH); (DMSO-d_6_, 75 MHz): δ116.30, 116.59, 120.60, 123.01, 129.10, 129.51, 130.07, 130.28, 130.40, 130.62, 131.04, 133.38, 134.44, 135.67, 137.34, 138.97, 150.22, 157.38, 162.57, 165.85, 167.48, 195.69; LC-MS (ESI):370. 2 (M-1).

***6-Benzoyl-2-(p-tolyl)quinoline-4-carboxylic acid (6c)***

Yield: 26%; mp=267-269 °C; IR (KBr): ν (cm^-1^) 2918.5(OH) 1700.7, 1653.7 (C=O); ^1^H NMR (300MHz-DMSO-d_6_):δ (ppm)7.38-7.40 (d, 2H, 4-methylphenyl H_3 _&H_5_, *J*=6Hz), 7.59-7.77(m, 3H, phenyl), 7.85-7.87 (d, 2H, 4-methylphenyl H_2 _&H_6_, *J*=6Hz), 8.13-8.16 (dd, 1H, quinolineH_7_, *J*=9Hz, *J*=2.5Hz), 8.22-8.27 (m, 3H, phenyl & quinoline H_8_), 8.24 (s, 1H, quinoline H_3_), 9.12-9.13 (d, 1H, quinoline H_5_, *J*=3Hz), 14.09 (s, 1H, COOH); (DMSO-d_6_, 75 MHz): δ21.43, 120.59,123.01,127.84, 127.90, 129.10, 129.55, 129.99, 130.15 ,130.27, 130.60, 133.35, 135.15, 135.48, 137.39,138.72, 150.36, 158.38, 167.55, 195.72; LC-MS(ESI): 366.0 (M-1)

***6-Benzoyl-2-(3,4-dimethoxyphenyl)quinoline-4-carboxylic acid (6d)***

Yield: 25%; mp=296-298 °C; IR (KBr): ν (cm-1) 2998.5(OH) 1719.5, 1649 (C=O); ^1^H NMR (300MHz-DMSO-d_6_):δ (ppm) 3.87(s, 3H, OCH_3_), 3.93(s, 3H, OCH_3_), 7.13-7.15 (d, 1H, 3&4-dimethoxyphenyl H_3_, *J*=6Hz), 7.61-7.64 (t, 2H, phenyl H_3_&H_5_, *J*=9Hz), 7.74-7.77(t, 1H, phenyl H_4_, *J*=9Hz), 7.85-7.88 (dd, 2H, phenyl H_2 _&H_6_*, J*=9Hz, *J*=2.5Hz), 7.92-7.95 (dd, 2H, 3&4-dimethoxyphenyl H_2 _&H_6_*, J*=9Hz, *J*=2.5Hz), 8.13-8.16 (dd, 1H, quinolineH_7_, *J*=9Hz, *J*=2.5Hz), 8.23-8.26 (d, 1H, quinolineH_8_, *J*=9Hz), 8.54(s, 1H, quinoline H_3_), 9.08-9.12 (s, 1H, quinoline H_5_), 13.0 (s, 1H, COOH); (DMSO-d_6_, 75 MHz): δ21.30, 21.73, 55.51, 56.66, 111.33, 111.57, 112.77, 120.23, 120.53, 122.72, 129.73, 129.91, 130.22, 130.49, 130.72, 135.17, 137.44, 138.81, 149.61, 150.31, 158.14, 172.47, 195.71; LC-MS (ESI):412.2 (M-1).

***General procedure for preparation of methyl 6-methoxy-2-arylquinoline-4-carboxylate***

2-arylquinoline-4-carboxylic acid (4 or 6) (2 mmol) and potassium carbonate (10 mmol) were mixed. Methyl iodide (10 mmol) and acetone (10 ml) were added. The reaction mixture was refluxed. The progress of the reaction was checked (TLC). The reaction was finished after 5 hr. The solvent was evaporated in vacuo and water was added to the residual mixture. The product was collected by filtration and dried to obtain pure product.

***Methyl 8-benzoyl-2-phenylquinoline-4-carboxylate (5a)***

Yield: 25%; mp=248-250 °C; IR (KBr): ν (cm-1) 1719.5, 1667.8 (C=O); ^1^H NMR (300MHz- CDCl_3_): δ (ppm) 4.01 (s, 3H, OCH_3_), 7.21-7.24 (t, 1H, phenyl H_4_ ,*J*=9Hz), 7.26-7.28 (d, 2H, phenyl H_3_&H_5_, *J*=6Hz), 7.31-7.36 (t, 2H, benzoyl H_3_&H_5_, *J*=9Hz), 7.44-7.47 (t, 1H, benzoyl H_4_*, J*=9Hz), 7.62-7.67 (m, 3H, quinoline H_6_& benzoyl H_2 _&H_6_), 7.7-7.73(d, 2H, phenyl H_2 _&H_6_*, J*=9Hz), 7.82-7.85(dd, 1H, quinoline H_5_*, J*=9Hz, *J*=2.5Hz), 8.34 (s, 1H, quinoline H_3_), 8.82-8.85 (dd, 1H, quinoline H_7_, *J*=9Hz, *J*=2.5Hz); ^13^C NMR (CDCl_3_, 75 MHz): δ 52.91 , 120.14 , 123.80, 127.25 , 127.84, 127.4, 128.23, 128.69, 129.56, 129.76, 12.67, 132.67, 135.56, 137.77, 139.16, 139.62, 146.88, 155.91, 166.60, 198.26; LC-MS(ESI): 368.2 (M+1), 391.2 (M+23).

***Methyl 8-benzoyl-2-(4-fluorophenyl)quinoline-4-carboxylate (5b)***

Yield: 72%; mp=183-185 °C; IR (KBr): ν (cm^-1^) 1721.49, 1659.43 (C=O); ^1^H NMR (300MHz-CDCl_3_):δ (ppm) 4.01(s, 3H, OCH_3_), 6.89-6.92(t, 2H, 4-flurophenyl H_3_&H_5, _*J*=9Hz),7.31-7.34 (t, 2H,4-flurophenyl H_2_&H_6_, *J*=9Hz),7.45-7.48 (t, 1H, phenyl H_4_, *J*=9 Hz), 7.59-7.65 (m, 3H, phenyl H_3_&H_5 _& quinoline H_6_), 7.68-7.71 (dd, 2H ,phenyl H_2_&H_6_, *J*=9Hz, *J=*2.5Hz), 7.83-7.86 (dd, 1H, quinoline H_5_, *J*=9Hz, *J*=2.5Hz), 8.29(s, 1H, quinoline H_3_), 8.81-8.84 (dd, 1H, quinoline H_7_, *J*=9Hz , *J=*2.5Hz);^ 13^C NMR (CDCl_3_, 75 MHz): δ 52.04, 115.31, 116.09, 119.60, 119.99, 123.68 , 127.68, 127.73, 128.04, 129.09, 129.87, 130.42, 133.98, 135.72 , 139.14, 139.46, 146.81, 154.83, 166.53, 198.23: LC-MS(ESI): 386.2 (M+1), 409.2 (M+23).

***Methyl 8-benzoyl-2-(p-tolyl)quinoline-4-carboxylate (5c)***

Yield: 78%; mp=178-180 °C; IR (KBr): ν (cm^-1^) 1726.01, 1665.83 (C=O); ^1^H NMR (300MHz- CDCl_3_):δ (ppm) 2.25(s, 3H, CH3), 4.0(s, 3H, OCH_3_), 7.02-7.04 (d, 2H, 4-methylphenyl H_3_&H_5_, *J*=6Hz), 7.30-7.33 (dd, 2H, phenyl H_3_&H_5_, *J*=9Hz, *J*=2.5Hz), 7.43-7.46 (t, 1H, phenyl H_4_, *J*=9Hz), 7.50-7.53 (dd, 2H, phenyl H_2_&H_6_, *J*=9Hz), 7.59-7.62(t, 1H, quinoline H_6_, *J*=9Hz), 7.70-7.73(dd, 2H, 4-methylphenyl H_2_&H_6_, *J*=9Hz, *J*=2.5Hz), 7.81-7.83 (dd, 1H, quinoline H_5_, *J*=6Hz, *J*=2.5Hz), 8.31(s, 1H, quinoline H_3_), 8.79-8.82 (dd, 1H, quinoline H_7_, *J*=9Hz, *J*=2.5Hz); ^13^C NMR (CDCl3, 75 MHz): δ 21.32, 52.88, 119.98, 123.67, 127.12, 127.15, 127.85, 128.20, 129.43, 129.53, 129.75, 132.61, 135.04 ,135.43, 139.25, 139.50, 140.25, 146.89, 155.87, 166.66, 198.37; LC-MS(ESI): 382.2(M+1), 404.2 (M+23)

***Methyl 8-benzoyl-2-(3,4-dimethoxyphenyl)quinoline-4-carboxylate (5d)***

Yield: 22%; mp=266-268 °C; IR (KBr): ν (cm^-1^) 1724.2, 1658.4 (C=O); ^1^H NMR (300MHz- CDCl3): δ (ppm) 3.48 (s, 3H, methoxy), 3.81 (s, 3H, methoxy), 4.02 (s, 3H, methoxy), 6.74-6.77(d,1H,3-4-dimethoxyphenyl H_3_, *J*=9Hz), 7.18-7.21(d, 1H, quinoline H_5_, *J*=9Hz), 7.28-7.47(m, 4H, phenyl H_3 _&H_4_&H_5_&3-4-dimethoxyphenyl H_2_), 7.61-7.64(t, 1H, quinoline H_6_*, J*=9Hz), 7.74-7.81(m, 3H, phenyl H_2 _&H_6_& 3&4-dimethoxyphenyl H_6_), 8.31(s, 1H, quinolineH_3_), 8.78-8.81(dd, 1H, quinoline H_7_, *J*=9Hz, *J*=2.5Hz);^ 13^C NMR (CDCl_3_, 75 MHz): δ 29.72, 55.69, 55.95, 109.78, 110.56, 119.78, 119.87, 123.41, 127.09, 127.71, 128.25, 129.44, 129.92, 130.66, 132.77, 135.42, 138.76, 139.50, 149.30, 150.91, 155.27, 166.68, 198.19; LC-MS(ESI): 428.2 (M+1), 451.2 (M+23).

***Methyl 6-benzoyl-2-phenylquinoline-4-carboxylate (7a)***

Yield: 52%; mp=145-147 °C; IR (KBr): ν (cm^-1^) 1729, 1649 (C=O);^1^H NMR (300MHz- CDCl3):δ (ppm) 3.93 (s, 3H, methoxy), 7.41-7.58 (m, 6H, benzoyl H_3_&H_4_&H_5_&phenyl H_3 _&H_4 _&H_5_), 7.82-7.84 (d, 2H, benzoyl H_2 _&H_6_, *J*=6Hz), 8.11-8.24 (m,4H, phenyl H_2_&H_6_&quinoline H_7 _&H_8_), 8.4(s, 1H, quinoline H_3_), 9.12(s, 1H, quinolineH_5_); ^ 13^C NMR (CDCl_3_, 75 MHz): δ52.94, 121.19, 123.02, 127.65, 128.43, 129.08, 129.32, 129.91, 130.28, 130.36, 130.66, 132.78, 136.16, 136.69, 137.35, 138.25, 158.73, 166.31, 196.11; LC-MS(ESI): 368.2 (M+1), 391.2 (M+23).

***Methyl 6-benzoyl-2-(4-fluorophenyl)quinoline-4-carboxylate (7b)***

Yield: 96%; mp=189-191 °C; IR (KBr): ν (cm^-1^) 1729, 1649 (C=O); ^1^H NMR (300MHz- CDCl3):δ (ppm) 3.93 (s, 3H, methoxy), 7.15-7.18 (t, 2H, 4-flurophenyl H_3 _&H_5_, *J*=9Hz), 7.46-7.59 (m, 3H, phenyl H_3_&H_4 _&H_5_), 7.81-7.84 (dd, 2H, phenyl H_2 _&H_6_ *, J*=9Hz, *J=*2.5Hz), 8.11-8.20 (m, 4H, quinoline H_7 _&H_8 _& 4-flurophenyl H_2 _&H_6_), 8.35(s, 1H, quinoline H_3_), 9.10 (s, 1H, quinoline H_5_);^ 13^C NMR (CDCl3, 75 MHz): δ29.73, 52.97, 115.93, 116.22, 120.76,120.50, 128.43, 129.29, 129.56, 129.67, 130.01, 130.26, 130.53, 132.80, 136.18, 136.79, 137.29, 157.51, 166.19, 196.02; LC-MS(ESI):386.2 (M+1), 409.2(M+23).

***Methyl 6-benzoyl-2-(p-tolyl)quinoline-4-carboxylate (7c)***

Yield: 74%; mp=187-189 °C; ^1^H NMR (300MHz- CDCl3):δ (ppm) 2.37 (s, 3H, 4-methylphenyl), 3.93 (s, 3H, methoxy), 7.27-7.29 (d, 2H, phenyl H_3 _&H_5_, *J*=6Hz), 7.46-7.49 (t, 2H, 4-methylphenyl H_3 _&H_5_, *J*=9Hz), 7.53-7.59 (t, 1H, phenyl H_3_*, J*=9Hz), 7.81-7.84 ( dd, 2H, phenyl H_2 _&H_6_, *J*=9Hz, *J*=2.5Hz), 8.05-8.08 (dd, 2H, 4-methylphenyl H_2 _&H_6_, J=6Hz, *J=*2.5Hz), 8.10-8.13 ( dd, 1H, quinoline H_7_, *J*=9Hz, *J*=2.5Hz), 8.19-8.22 ( d, 1H, quinoline H_8__, _*J*=9Hz), 8.38 (s,1H.quinoline H_3_), 9.11 (s, 1H, quinoline H_5_); ^ 13^C NMR (CDCl_3_, 75 MHz): δ 21.44, 52.90, 121.03, 121.73, 122.86, 127.52, 128.40, 129.33, 129.79, 129.82, 130.25, 130.51, 132.73, 135.42, 135.89, 136.56, 137.37, 140.69, 158.68, 166.36, 196.14; LC-MS(ESI): 382.2 (M+1), 405.2 (M+23).

***Methyl 6-benzoyl-2-(3,4-dimethoxyphenyl)quinoline-4-carboxylate (7d)***

Yield: 91%; mp=193-195 °C; IR (KBr): ν (cm-1) 1724.2, 1658.4 (C=O); ^1^H NMR (300MHz- CDCl_3_):δ (ppm) 3.85 (s, 3H, methoxy), 3.89 (s, 3H, methoxy), 3.99 (s, 3H, methoxy), 6.91-6.94 (d, 1H, 3&4-dimethoxyphenyl H_3_, *J*=9Hz) , 7.43-7.48 ( t, 2H, phenyl H_3 _&H_5_, *J*=9Hz), 7.56-7.59 (t, 1H, phenyl H_4_, *J*=9Hz) , 7.66-7.69 ( dd, 1H, 3&4-dimethoxyphenyl H_3, _*J*=9Hz, *J=*2.5Hz), 7.81-7.86 (m, 3H, phenyl H_2 _&H_6 _&3-4-dimethoxyphenyl H_6_), 8.10-8.13 (dd, 1H, quinoline H_7__, _*J*=9Hz, *J=*2.5Hz), 8.18-8.21 (d, 1H, quinoline H_8_, *J*=9Hz), 8.35 (s, 1H, quinoline H_3_) , 9.08 (s, 1H, quinoline H_5_); ^ 13^C NMR (CDCl_3_, 75 MHz): δ 52.92, 56.05, 56.13, 110.27, 111.06, 120.74, 120.82, 122.67, 128.40, 129.41 , 129.86, 130.24, 130.35, 131.01, 132.72, 135.71, 136.53, 137.39, 149.59, 150.68, 151.29, 158.15, 166.41, 196.13; LC-MS(ESI): 428.2 (M+1), 451.2 (M+23)

***Biological assays***

*Cytotoxicity assay*

The MTT assay was done by seeding 5.0×10^3^ human cancer cells per well in 96-well plates (32-41). Following overnight incubation of the cells in 5% CO_2_ at 37^°^C, culture medium of each well was exchanged with medium having reference anticancer drug, cisplatin (0-100 µM) or different concentrations of newly synthesized quinolines (0-100 µM) or ketoprofen. Then cells were incubated for 72 hr. MTT solution (25 μl, 4 mg ml ^-1^) was added to each well and the cells were incubated at 37 °C for 3 hr. Finally, formazan crystals were dissolved in DMSO (100 μl) and absorbance was read in a plate reader (Synergy H4, USA) at 540 nm. 

*MDR reversal studies*

The MTT based assay was done by seeding 5000 cancer cells per 180 µl RPMI complete culture medium in each well of 96-well culture. Cisplatin was applied at concentrations of 12.5, 25, 50 and 100 μM in both A2780 and A2780/RCIS cancer cells in absence or presence of highest non-toxic concentrations of synthetized compounds. Cells were then incubated (37 ºC in 5% CO_2_ incubator) for 48 hr. Then 25 µl of MTT solution (4 mg ml^-1^) were added to each well and then incubated at 37 ºC (3 hr). At the end of incubation, formazan crystals were dissolved in DMSO (100 µl) and plates were read in a plate reader (Synergy H4, USA) at 540 nm. This experiment was done in triplicate determination each time. 

*Flow cytometric efflux assay*

 Microplates containing1×10^6 ^resistant cells in each well were incubated with 10 μM of 5-CFDA for 60 min. After washing, synthetized compounds were added and the cells were further incubated (60 min). Cells were washed with ice-cold PBS (two times) and harvested. After centrifugation, supernatants were removed and cells suspended in ice-cold PBS. Samples were analyzed by a BD FACS Calibur Flow Cytometer (BD Biosciences, San Jose, USA). Fluorescence intensity of substrate accumulated in the cells was measured with FlowJo 7.6.1 data analysis software (Oregon, USA). Cells treated with ketoprofen were used as controls.

*Molecular modeling*

Mode of interaction between synthesized ligands and homology modeled ABCC2 (MRP2) was investigated by docking. 2D structure of chemicals was organized in Chem Draw Ultera 12.0 software and 3D structures were arranged by Chem Draw Ultra 12.0 software using molecular mechanic force filed pre-optimization monitored by MM2 calculation. Further modification such as polar hydrogen addition was achieved by MOE software. Synthesized chemicals were docked into the binding site of MRP2 by MOE software. The docking simulations were done using triangle matcher placement algorithm with London dG scoring function and force field as refinement method. For each compound, the top-score docking poses were selected for final ligand–target interaction analysis using LigX module in MOE Software. 

## Results

***Synthesis***

**Scheme 1:**
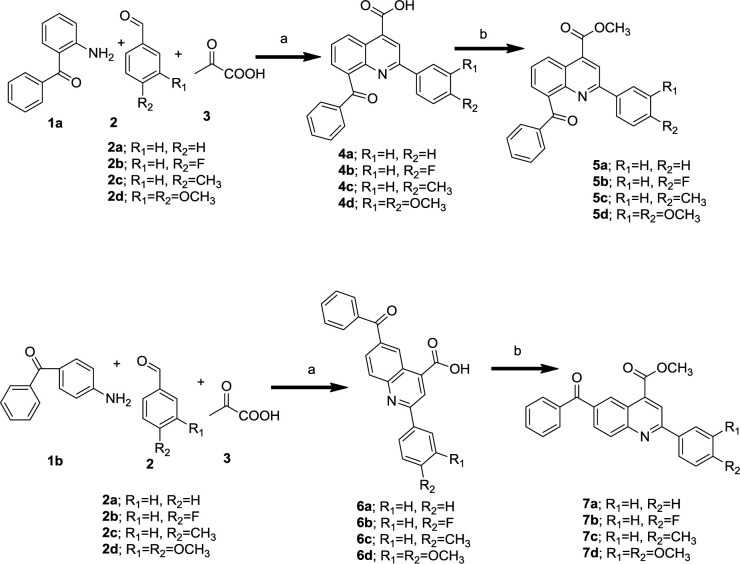
Reagents and conditions: (a) acetic acid, reflux (b) K_2_CO_3_, CH_3_I, Acetone, reflux

A one-step Doebner reaction was used to make 2-arylquinoline-4-carboxylic acid derivatives. As shown in scheme 1, 2- or 4-aminobenzophenone **1**, substituted benzaldehyde **2 **and pyruvic acid **3** were refluxed in acetic acid to obtain 4-carboxy quinolines (**4** and **6**) (42) and then esterification of carboxylic acid group was performed using methyl iodide in acetone (43) to afford the novel quinoline-4-methyl esters (**5 **and **7**). The compounds were characterized by nuclear magnetic resonance, infrared spectroscopy and mass spectroscopy.

***Biological evaluation***

*In vitro cytotoxic effects*

Mahdizadeh* et al. *(44) examined the basic level of the mRNA expression of MRP1 and MRP2 in A2780/RCIS cells and sensitive parental A2780 cell line. They reported that the MRP1 mRNA level in the resistant cell line (A2780/RCIS) was 1.29 times more than its expression level in sensitive cells (A2780 cells). Also, their results displayed that the expression level of MRP2 mRNA in the A2780/RCIS (resistant cell line) was much more (13 times) than the MRP2 mRNA level in parental A2780 cells. To identify ideal MRP inhibitors reversing MDR at non-toxic concentrations, cytotoxicity of the quinoline compounds against parental sensitive A2780 cells and their resistant sublines A2780/RCIS cells which overexpress MRP2 was evaluated by MTT assay. Cisplatin and ketoprofen were selected as controls. Most of our compounds exhibited negligible or much lower cytotoxic effect in both cancer cells. As depicted in Table 1, four quinoline derivatives **5a**,** 6b**,** 6c **and** 7b** showed moderate cytotoxic activity with IC_50_ in the range of 31.95-84.41 μM. However, the other quinolines did not display cytotoxic activity at concentrations below 100 μM.

*Reversal of MRP -mediated MDR by quinoline derivatives*

The reversal of multidrug resistance by the new quinoline derivatives was evaluated in drug-resistant cancer cell line with overexpression of MRP2 (A2780/RCIS). The multidrug resistant cancer cell lines are remarkably resistant to the corresponding substrate anticancer drugs. We determined the cytotoxicity of cisplatin, in A2780/RCIS, multidrug resistant ovarian carcinoma cells (MRP2-overexpressing ovarian carcinoma cell line) and A2780, drug-sensitive ovarian carcinoma cells. The resulting IC_50_ values are shown in Table 2. Our compounds are two groups, the first group is 8-benzoyl quinoline derivatives and the second group which is the isomers of the first group is 6-benzoyl quinoline derivatives. Compounds **4c**,** 5a**,** 5b** and **5c **from the first group and** 6d**,** 7a**,** 7b **and** 7d** from the second group at 30 μM concentration (almost the highest common non-toxic concentration between all synthetized compounds) exerted MDR reversal, and increased the anticancer activity of cisplatin in the human MRP2 overexpressing cell line A2780/RCIS. Compound **7d** from the second group possessing dimethoxy phenyl in position 2 of quinoline exerted the most MDR reversal activity, and enhanced the cytotoxicity of cisplatin more than the other quinolines. 

*Biological evaluation of the MRP2 inhibition*

Compounds exerted MDR reversal, and enhanced the cytotoxicity of cisplatin in the human MRP2 overexpressing cell line A2780/RCIS, including **4c**,** 5a**,** 5b**, **5c **(from the first group),** 6d**,** 7a**,** 7b **and** 7d** (from the second group) were selected to investigate their MRP2 inhibition activity. MRP2 inhibition was evaluated by the determination of the uptake amount of the fluorescent 5-carboxy fluorescein diacetate (5-CFDA) substrate, by A2780/RCIS ovarian carcinoma cells overexpressing MRP2 in the presence of the selected compounds. Compounds from the first group **4c**,** 5a**,** 5b**, **5c **did not show significant MRP2 inhibitory activity at the concentration below 200 μM. Compound **4c** showed the most potent MRP2 inhibitory activity in the first group in concentration of 500 μM in a dose-dependent manner (data not shown). 

When compounds from the second group tested at the concentration of 30 μM, none of the compounds except **6d** and **7d** were found to inhibit the efflux of 5-carboxyfloresin diacetate in A2780/RCIS cells (data not shown) compound **6d,** a 4-carboxy quinoline possessing dimethoxy phenyl in position 2 of quinoline ring, showed the most potent MRP2 inhibition among all the tested quinolines in a dose-dependent manner and more than the reference drug ketoprofen. Surprisingly, compound **7d** which exerted the most MDR reversal, and enhanced the cytotoxicity of cisplatin more than the other quinolines did not show the most MRP2 inhibition activity. 

*Docking studies*

As X-ray crystallization of the MRP2 protein is not accessible, the lone structural information existing to date is a bacterial ABC transporter. A previous structure of a bacterial MDR-ABC transporter MsbA has been remoted due to incorrect topological assignments resulted from low resolution of the X-ray diffraction data (45). However, using the obsolete PDB entry a homology model of ABCC2/MRP2 has been built to predict the binding of our quinolines.

*Homology modeling of ABCC2 (MRP2)*

The 1545-amino acid human ABCC2 (MRP2) contains two nucleotide binding domains (46) and up to 17 transmembrane helices distributed within three transmembrane domains (TMD), 1, 2, and 3. It has been shown that the amino terminal TMD1 of ABCC1 is not essential for substrate transport. So experiments have focused on TMD2 and 3. Sequence for ABCC2 had swiss port entry Q92887. As it was described previously (47) we used lipid flippase MsbA (chain A and B) with Data Bank entry: 1pf4 as a model for TMD-2 and 3. We used MOE2019 for homology modeling with its default settings. We used residues Lys329, Met 440, Ser 444, Gln 447, Ile 476, Ile 479, Gln 543, Cys 544, Val 546, Phe 550, Thr 553, Val 557, Ser 558, Phe 562, Asn 587, Ile 588, Leu 589, Arg 591, Met 595, Met 598, Met 599 as binding site. MOE2019 with its default setting was employed for docking studies (Figure 3).

To explain the results of biological experiments docking studies of ketoprofen, compounds **7d** and **6d** into the homology-modeled human MRP-2, were carried out (Figure 3). As mentioned above, compound **6d,** a 4-carboxy quinoline possessing dimethoxy phenyl in position 2 of quinoline ring, showed the most potent MRP2 inhibition among all the tested quinolines in a dose-dependent manner and more than the reference drug ketoprofen. Studying ligand interaction mode of **6d** by LigX module of MOE software revealed that O and H atoms of carboxyl group of **6d**, could form hydrogen bonds with MET 595 and MET 598 (Figure 4). The O atom of benzoyl group made hydrogen bond with ARG 393. Methoxy groups of **6d **can made contact with the backbone of several amino acid residues, like Phe 591 and Phe 550. Compound **7d** methyl ester of **6d**, interacted less compared to its parent **6d**. As shown in Figure 4, the O atom of benzoyl group of **7d** made hydrogen bond with ARG 393, the same as that of **6d**, but esterification of **6d** led to eliminate the hydrogen bonds with MET 595 and MET 598. Ketoprofen also interacted less than its derivatives **6d** and **7d**. As shown in Figure 4, the O atom of benzoyl group of ketoprofen made hydrogen bond with ARG 393, the same as that of **6d** and **7d**, O atom of hydroxyl group of ketoprofen, could form hydrogen bonds with MET 595, the same as **6d**. Although ketoprofen possess carboxyl group which forms hydrogen bond with target, but its binding energy is more than its derivatives **6d **and **7d** (Table 3), indicating that the quinoline ring causes the carboxyl group to be placed in a direction that can interact more with the target, and also dimethoxy phenyl ring provided additional interactions with the target. 

**Figure 1:**
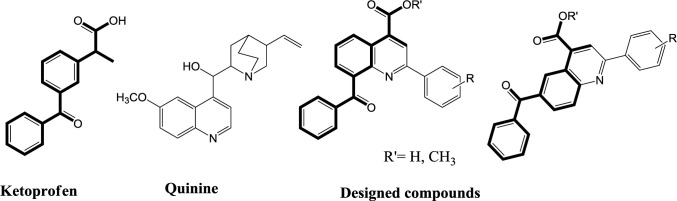
Chemical structures of ketoprofen, quinine and our designed quinoline derivatives possessing ketoprofen scaffold as MRP2 inhibitors

**Figure 2:**
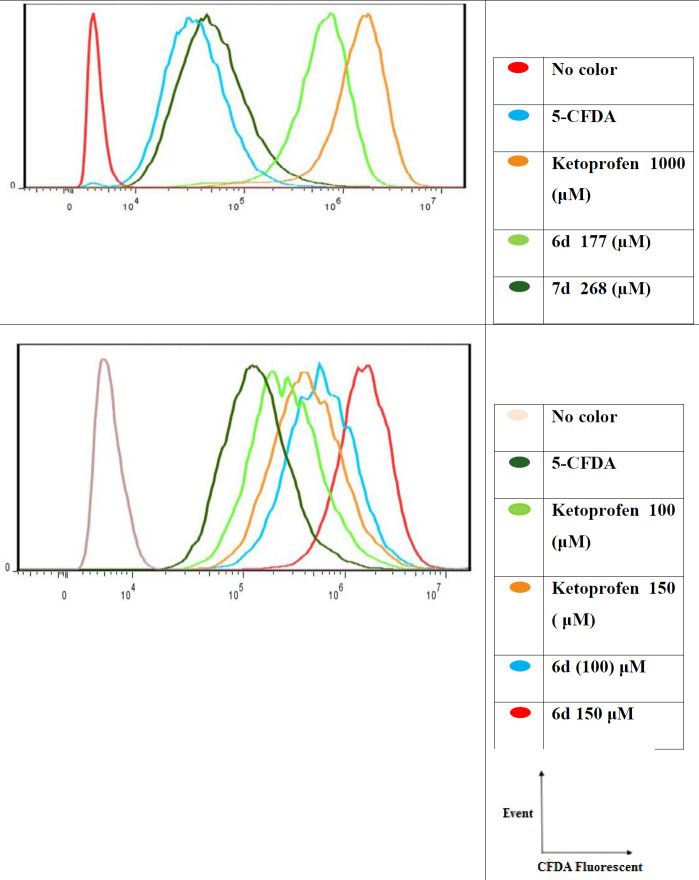
The uptake amount of the fluorescent 5-carboxy fluorescein diacetate (5-CFDA) substrate, by A2780/RCIS in the presence of compounds 6d, 7b, 7d and ketoprofen

**Table 1:** The *in vitro* antiproliferative activities of quinolines, ketoprofen and cisplatin against A2780 (drug-sensitive ovarian carcinoma cells) and A2780/RCIS (multidrug resistant ovarian carcinoma cells)

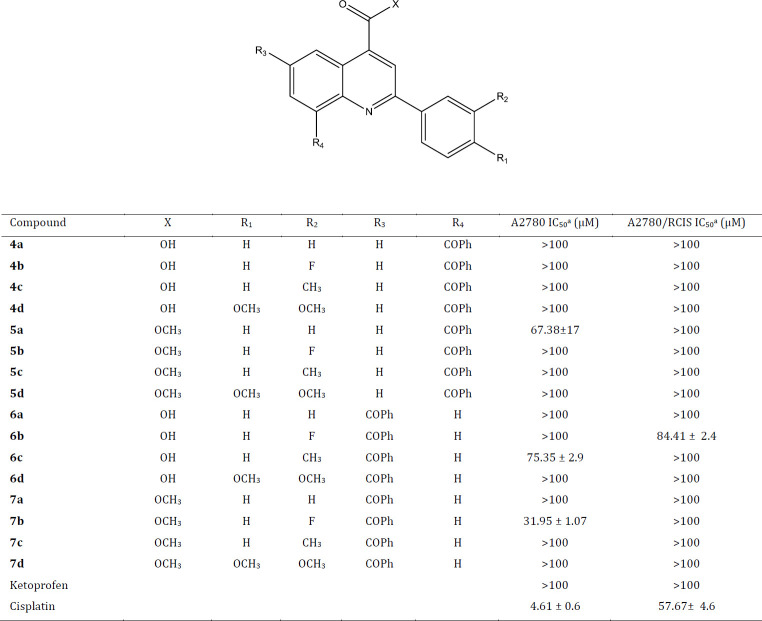

^a ^Compound concentration required to inhibit tumor cell proliferation by 50%. Data are presented as the mean ± SD from the dose−response curves of three independent experiments

**Table 2:** the cytotoxicity of cisplatin, in A2780/RCIS, multidrug-resistant ovarian carcinoma cells (MRP2-overexpressing ovarian carcinoma cell line) alone or in the presence of compounds

Compound	A2780/RCIS IC_50_^a^ (μM)	Compound	A2780/RCIS IC_50_^a^ (μM)
**Cis +4a**	53.62±3.45	**Cis +6a**	64.55±1.7
**Cis +4b**	63.21±2.96	**Cis +6b**	62.84±2.1
**Cis +4c**	37.34±3.87	**Cis +6c**	56.27±3.8
**Cis +4d**	52.67±4.36	**Cis +6d**	35.54±0.9
**Cis +5a**	45.23±4.12	**Cis +7a**	48.06±0.51
**Cis +5b**	48.34±3.35	**Cis +7b**	25.17±1.6
**Cis +5c**	28.56±5.34	**Cis +7c**	ND
**Cis +5d**	54.34±3.95	**Cis +7d**	14.88±1.1
**Cisplatin **	57.67±4.61		

**Figure 3:**
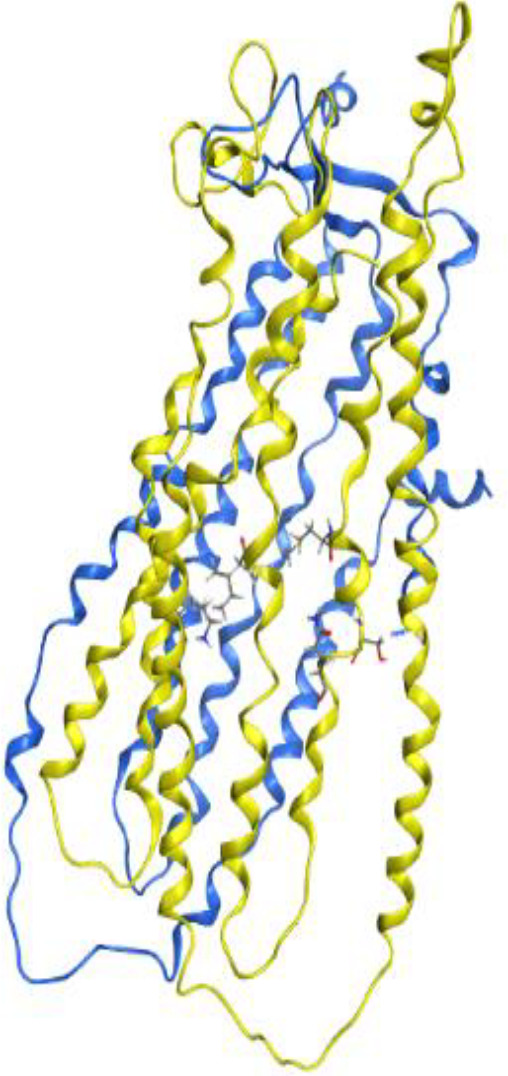
TMD2 (blue) and TMD3 (yellow) model for ABCC2

**Figure 4:**
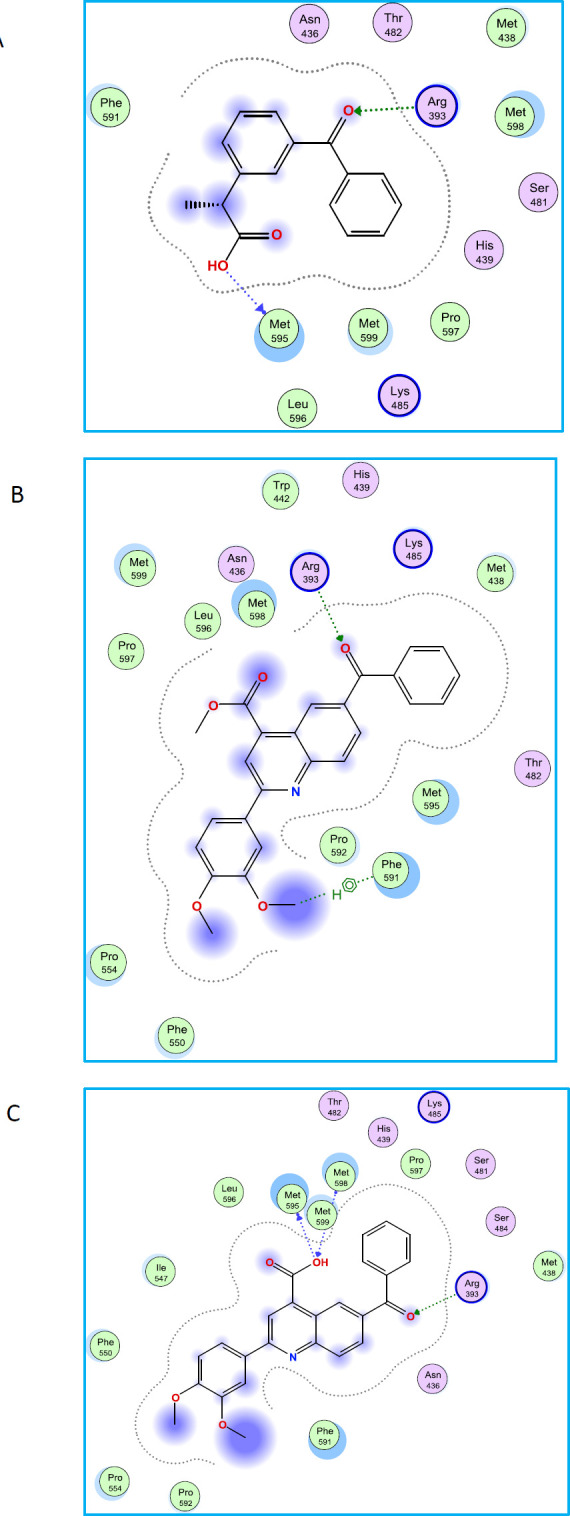
The 2D representation of the interaction between ketoprofen (A), compounds **7d** (B) and **6d** (C) in the homology modeled ABCC2 (MRP2) using LigX in MOE

**Table 3:** Results of molecular docking experiments for ketoprofen, compounds **7d** and** 6d**

**Compounds**	**AutoDock binding** **energy (kcal/mol)**	**Residue**	**Ligand atoms**	**Distance (Å)**	**Interaction**
ketoprofen	-6.68	MET 595	OH	2.57	Hydrogen bond
		ARG 393	CO	2.74	Hydrogen bond
**7d**	-7.79	ARG 393	CO	2.79	Hydrogen bond
		PHE 591	C	4.88	H-pi
**6d**	-8.67	MET 595	OH	2.53	Hydrogen bond
		MET 598	OH	3.21	Hydrogen bond
		ARG 393	CO	2.77	Hydrogen bond

## Discussion

This study indicates that 6- or 8-benzoyl-2-arylquinoline is a suitable scaffold (template) to design MRP2 inhibitors. The position of benzoyl in quinoline ring is important in inhibition of MRP2. Generally, 8-benzoyl-2-arylquinolines showed more activity compared to their isomers (6-benzoyl-2-arylquinolines). Compound **6d**, a 4-carboxy quinoline possessing dimethoxy phenyl in position 2 of quinoline ring, showed the most potent MRP2 inhibition among all the tested quinolines in a dose-dependent manner and more than the reference drug ketoprofen. MRP2 inhibition activity of compound **7d** was less in comparison to that of **6d**, indicating that carboxyl group in position 4 of quinoline may interact with MRP2. These hydrophobic interactions and hydrogen bonds formation of compounds with homology modeled MRP2 can describe inhibitory effect of these compounds. Docking studies showed that compound **7d** methyl ester of **6d**, interacted less compared to its parent **6d**, which is consistent with biological results.

## Conclusion

Benzoyl-2-arylquinoline is a suitable template to design MRP2 inhibitors. The position of benzoyl in quinoline ring is important in inhibition of MRP2. Carboxyl group in position 4 of quinoline may interact with MRP2. Docking studies described the biological results and is consistent with biological results.
